# High-intensity training on CREB activation for improving brain health: a narrative review of possible molecular talks

**DOI:** 10.3389/fendo.2024.1498495

**Published:** 2025-01-20

**Authors:** Ping Li, Yan Hu, Ligang Tong, Xuecui Bi

**Affiliations:** ^1^ Faculty of Sports Science, Ningbo University, Ningbo, China; ^2^ Ningbo High-tech Zone Playing Kindergarten, Ningbo, China; ^3^ Xianjiang Honors School of Arts and Physical Education, Ningbo Childhood Education College, Ningbo, China; ^4^ Physical Education Institute, Capital University of Physical Education and Sports, Beijing, China

**Keywords:** CREB, HIT, physical exercise, neurons, brain health

## Abstract

Although physical exercise has obvious benefits in brain physiology, the molecular biomarkers induced by exercise protocols are inconclusive. Evidence indicates that exercise interventions are effective in shaping brain physiology. However, the potential mediator for improving brain functions is uncertain. CREB is one of the potential targets of exercise that triggers various molecular cross-talk to improve neurogenesis, long-term potentiation, and synaptogenesis. Therefore, CREB may be situated on the causal path between maintaining brain health and exercising. To support this, studies have shown that exercise-mediated CREB phosphorylation improves cognitive functions and memory. In addition, among the protocols of exercise (types, duration, and frequency), the intensity has been reported to be the most effective in triggering CREB-mediated molecular signaling. For example, HIT increases the synthesis of CREB, which may not only induce brain physiology but also induce brain pathology by higher activation of its downstream targets, such as BDNF. Therefore, this review aims to understand the effects of HIT on CREB function and how HIT can mediate the CREB-induced molecular cross-talk for maintaining brain health.

## 1 Introduction

Maintaining brain health and cognitive function impacts the quality of life as individuals age. In recent years, there has been growing interest in finding lifestyle factors, including physical exercise as a neuroprotector for improving brain health (1, 2). However, the uncertainty of physical exercise protocols to trigger neuroprotective effects renders physical exercise as major lifestyle factor in reversing neural decline and following benefits (1). Therefore, finding a specific exercise protocol and underlying neuroprotective mechanism could reveal the importance of physical exercise. A well-programmed exercise protocol activates several genes and molecular pathways, including *PPARGC1A* encoding peroxisome proliferator-activated receptor gamma coactivator 1-alpha (PGC-1α), pyruvate dehydrogenase kinase 4 (*PDK4*) and myocyte enhancer factor 2A (*MEF2A*) (1–3). These pathways then positively affect every cell and tissue, even at the systemic levels, evidenced by mitochondrial biogenesis, increased capillarization, vascular adaptation, and cell survival (3, 4). However, these effects are based on exercise intensity, duration, and individual genetic makeup (5). Therefore, designing exercise protocols is always in the interest of people and athletes who want to perform regular exercise and even researchers who want to understand the exercise-induced benefits.

Designing specific exercise protocols has been broadly categorized into two types: aerobic training with 50-80% of VO2 max or high-intensity training (HIT) with over 90% of VO2 max (3). Nevertheless, HIT has slight advantages over traditional exercise types as it produces more adaptive responses and increases the threshold capacity of individuals in a shorter time, mediated by several molecular cross-talks and subsequent activation signal transduction such as mitogen-activated protein kinase (MAPK), protein kinase C (PKC) and AMP-dependent protein kinase (AMPK) (6). Emerging evidence suggests that HIT can enhance cerebral blood flow (5), increase the production of neurotrophic factors (2), and promote neurogenesis (5), all of which may contribute to improve cognitive function and neuroprotection (2). Additionally, HIT has been associated with reductions in systemic inflammation by reducing c-reactive protein, IL-1β, and IL-10 (5), a key factor linked to neurodegeneration. However, most of these studies reported HIT-induced benefits among young and healthy people (6). Therefore, further research is required to establish the effects of HIT on elderly and diseased populations. Consequently, it prevents non-communicable lifestyle diseases, including metabolic and neurodegenerative diseases, among aged people (6). cAMP response element-binding protein (CREB) is one molecular protein activated directly by exercise or exercise-induced upstream targets of CREB. Consequently, it organizes the molecular cross-talk with molecular up and downstream targets. Therefore, this review discusses the possible role of HIT in activating CREB through various molecular cross-talks. CREB-like proteins are crucial for designing brain architecture by restoring neuronal signals and preventing local neuronal loss (7). Cell survival signals such as phosphatidylinositol 3-kinase/protein kinase B (PI3/AKT) and mitogen-activated protein kinases (MAPKs) converge on the CREB family to improve neuronal health (7), and these signaling pathways are stimulated by exercise (3). However, exercises like HIT regimens keep these signals long-lastingly activated (5); thus, CREB-mediated transcriptional programs induce many genes related to brain health, including synaptic plasticity, proliferation, differentiation, and improved cognitive functions (3). For instance, the HIT-activated sympathetic nervous system releases stress hormones like catecholamines and subsequent protein kinase A activation (PKA) for CREB phosphorylation (8). This can trigger long-term potentiation (LTP) for memory formation (9). However, all these signals can also be attributed to neuronal loss and induce neurodegeneration (3). Therefore, it is important to understand how HIT orchestrates the molecular events that induce CREB to improve brain health.

## 2 The basic structure of CREB

CREB was originally isolated from the brain of a rat (10). It stimulates the transcription of the somatostatin gene in response to the flux of cellular cAMP levels. Both animals and humans have CREB genes composed of 11 exons, having a molecular weight of 43 kDa. Also, it has three different isoforms functionally indistinguishable in all tissues. It belongs to the family of leucine zipper transcription factors and requires serine phosphorylation of CREB residue at the 133rd position to activate itself (11). PKA is the first kinase to activate the CREB in response to the increase of cAMP level. Activating serine phosphorylation could bring the CREB binding protein (CRB) to CREB (12). Acetylation of CREB can also increase its transcription functions along with other external stimuli through various molecular pathways, such as MAPKs, Calmodulin-dependent protein kinases (CaMK) I, II, and IV, and Akt (13).

## 3 Methodology

To explore how HIT affects the CREB functions in the brain, a literature search was done related to the topic from December 2023 to September 2024 using different scientific databases, including PubMed, Google Scholar, and Web of Science from October 1998 to September 2024. The focus was to identify the mechanism of CREB that can cross-talk with other molecular signaling during HIT exercise. For that, specific keywords were employed (Medical Subject Headings [MeSH] terms) related to “ Physical exercise AND CREB”, “ High-intensity exercise AND CREB”, “upstream and downstream targets of CREB and High-intensity exercise”, CREB induced genes and high-intensity exercise”, Different types of HIT and CREB expression. These keywords were combined with Boolean operators (AND and OR) to select the articles that directly focused on HIT and CREB functions in the brain. The selection procedure began with reviewing the titles, followed by the abstracts, and then the full texts. Duplicate articles were identified and removed after a careful evaluation of the articles of the titles by each author. A total of 401 articles were involved in the selection process. From these, we removed 320 articles after a preliminary assessment of the titles and abstracts. Subsequently, 68 more articles were excluded after a full-text screening. Finally, five articles that met the criteria and were relevant to the topic. However, we excluded five articles as they were assessed CREB from skeletal muscle and two articles were removed due to protocols used (low and medium intensity exercise on CREB function in the brain). Finally, we included six articles to identify the possible molecular talks of CREB upon HIT in the brain.

## 4 Results

Study details are given in **Supplementary Table 1** (**Supplementary File**), consisting of 7 studies, which all followed running HIT protocols to assess the effect of CREB in improving brain functions. Lei et al. showed that an increase of lactate during HIT affects brain function in aging by increasing CREB and causing metabolic flux in the hippocampus (11). During this scenario, several molecular cross-talks were organized by CREB, including AKT, HSL, LDH, PGC-1 alpha, SIRT1, and BDNF (11). Wu et al. showed that CREB expression was increased by mutually activating BDNF in the hippocampus in the feedback loop mechanism and negatively affected brain functions by deteriorating spatial learning and memory (14). Aguiar et al. showed that HIT increased the phosphorylation of the Ser-133 site to activate CREB in the hippocampal region rather than the cortex and striatum to affect memory (15). Mojtahedi et al. showed that HIT protocols did not increase the CREB activity in the hippocampus when compared to low and voluntary running exercise (16). Shen et al. showed that the HIT group increased spatial memory by slightly increasing CREB in the hippocampus (17). Jin et al. showed that swimming exercise with 60 mins for 6 weeks with HIT increased the PDE4 methylation for activating cAMP/PAK/CREB mediated signaling in the hippocampus (18). Zhang et al. reported that 7 weeks of HIT protocols decreased the CREB phosphorylation, and had negative effects on hippocampal plasticity (12).

## 5 Effect of HIT on CREB induced molecular signaling in cognitive function

Targeting exercise-mediated proteins can trigger CREB-mediated transcriptional program, thus, improving cognitive functions. For example, phosphodiesterase 4 (PDE4) is one of the targets of exercise that activates CREB (19). HIT, in a longer duration, increases the concentration of cAMP to consolidate learning and memory by improving cAMP-PDE4 interactions (19). Six weeks of swimming exercise in HIT protocols induce the PED4 methylation and activate cAMP/PAK/CREB for synaptic transmission, excitability, and plasticity of neurons to produce neuroprotection in the hippocampus (20). In addition, HIT-induced activation of cAMP/PKA/CREB triggers the brain-derived neurotrophic factor (BDNF) production for BDNF-mediated cognitive functions (20). Moreover, HIT exercise-induced β-adrenergic receptor and calcium flux from the skeletal muscle triggers the CREB-regulated transcription coactivator 1 (CRTC1) that stimulates the genes involved in learning and memory (21, 22). HIT Swimming exercise increases the activation of CBP and histone acetylation in the hippocampus to improve cognitive functions by increasing LTP and long-term memory formation (23, 24). HIT resistance training with a power of 85% of maximum repetition increases the MAPK/ERK and c-Jun N-terminal kinases (JNK) (25), which can induce the binding of CREB on the promotor region of BDNF (26). Also, resistance training with high intensity increases the release of muscle BDNF, which can bind to brain tropomyosin receptor kinase B (TrkB) to activate different CREB-mediated signaling cascades such as PI3/AKT/mTOR, Ras/MAPK/ERK, and phospholipase Cγ (PLCγ)/CamKII/CREB (26). This can promote the additional secretion of BDNF in the brain to increase CREB-mediated cognitive functions (27). HIT-induced increase of Arc, c-Fos, and EGR improve the neuroplasticity and cognitive functions, learning, and memory through interacting with CREB (28).

## 6 The metabolic control of CREB for brain health- role of HIT

**Figure 1:**
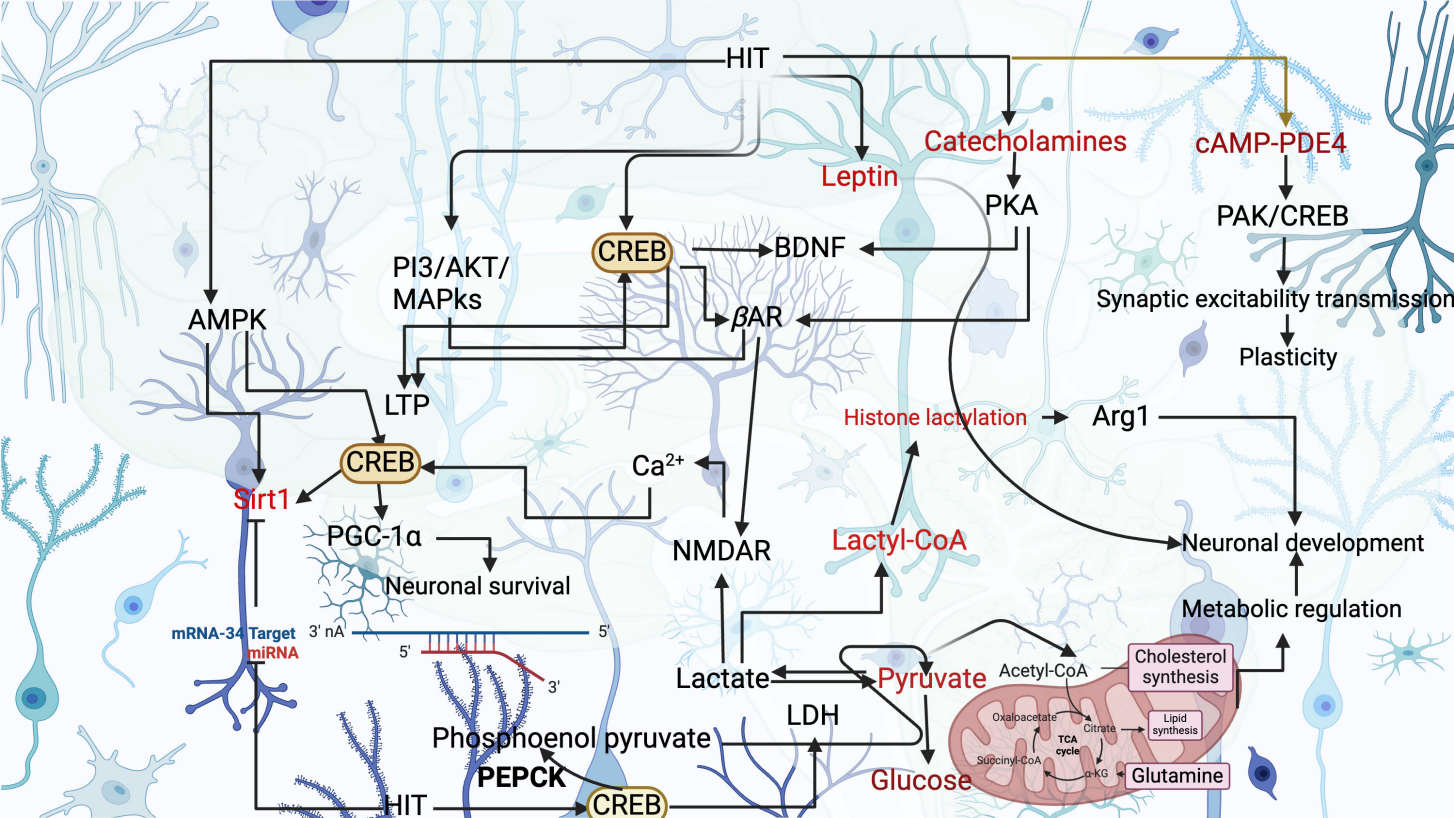
High-intensity training (HIT) downregulates the miR-34a for SIRT1 expression, possibly mediated by AMPK and CREB. HIT-induced CREB improves the LDH ratio for lactate metabolism, which can synthesize lactyl-CoA and histone lactylation for neuronal development via stimulating Arg1 gene. This scenario induces NMDAR and Ca2+ flux for CREB expression in a feedback manner. HIT-induced catecholamines can also activate NMDAR for CREB expression. HIT-triggered cAMP-phosphodiesterase 4 (PDE4) activates PAK/CREB for synaptic excitability transmission and plasticity.

CREB has a crucial function in metabolic health by triggering metabolic genes. For example, Sirtuin 1 (Sirt1), a metabolic sensor, regulates energy homeostasis within the brain. However, its activity is post-translationally modified by the miR-34a for neuronal plasticity and memory formation, mainly by CREB-mediated mechanisms in the brain (29–31). However, overexpression of miR-34a triggers cognitive impairment rapidly (32). In this scenario, HIT downregulates the miR-34a through CREB phosphorylation and affects the Sirt1 function (33). Thus, it can rewire the metabolic functions in the neurons (33). The N-methyl-D-aspartate receptor (NMDAR) is another crucial HIT-induced glutamate receptor present in neurons, which plays a crucial role in neuronal health by improving metabolic homeostasis (34). Nevertheless, excessive activation of NMDAR disregulates the metabolism and causes brain injury (34). Studies have shown that exercise training increases the activity of NMDAR (35, 36), which improves the opening conductance level in cerebral infarction and accelerates LTP in the hippocampal area (37). Moreover, HIT-induced lactate accumulation may increase the activity of NMDAR and disrupt the initial metabolic adaption in the brain (24, 38). This metabolic demand activates the CREB signaling in the cortex and improves the metabolic-dependent plasticity in the hippocampal area (39). Establishing the link between HIT-induced lactate accumulation and NMDAR and CREB role requires further clarification because lactate accumulation shifts the LDH ratio and causes brain aging, and CREB phosphorylation induces LDH transcription (40) (**Figure 1**). In addition, lactate plays a crucial role in increasing BDNF-induced benefits such as neurogenesis, neuronal survival, synaptic plasticity, and dendritic spine growth (41), and lactate can also serve as an important metabolic source in the neurons (42). Initial redox flux due to HIT may induce a short time of metabolic disruption in the brain (43). Byproducts like H2O2 from this scenario increase the CREB phosphorylation through epidermal growth factor receptor (EGFR) and MAPK (44, 45). HIT protocols increase the glycolytic enzymes for improving brain metabolic functions. For example, twelve weeks of HIT running at 90% of maximum heart rate increases the hexokinase 2 (13), which is mediated by HIT-induced CREB phosphorylation that activates the hexokinase transcription for regulating glycolysis in the astrocytes of the brain (13). CREB can also activate gluconeogenic genes, including phosphoenolpyruvate carboxykinase (PEPCK) and glucose-6-phosphatase (46), crucial for neuronal steroidogenesis during acute inflammation in the brain (47). This scenario is mediated by the hormonal flux of HIT, such as glucagon, catecholamines, and glucocorticoids (48), which can increase the gluconeogenic flux and improve the metabolic health of the neurons (49). A study has shown that HIT may have a negative effect on CREB, which is downregulated by 7 weeks of HIT (5 sessions per week, at 90% V_peak_) (37) in the hippocampus, while another study has shown that 85% max speed for 7 weeks improved the CREB signaling in the hippocampus (50), possibly the duration and intensity play a greater role in increasing CREB in the hippocampus (50). Other signaling pathways, such as AMP-activated protein kinase (AMPK), p38MAPK, and peroxisome proliferator-activated receptor gamma coactivator 1-alpha (PGC-1 α), can also be triggered by HIT and induce mitochondrial biogenesis through CREB signaling pathways (51). This can improve the metabolism of glucose and fat in the brain (51). In addition, CREB may remodel the endothelial functions through the activation of p38MAPK, which is crucial for angiogenesis in the central nervous system (52).

## 7 CREB’s role on neuronal development- different types of HIT protocols

**Figure 2:**
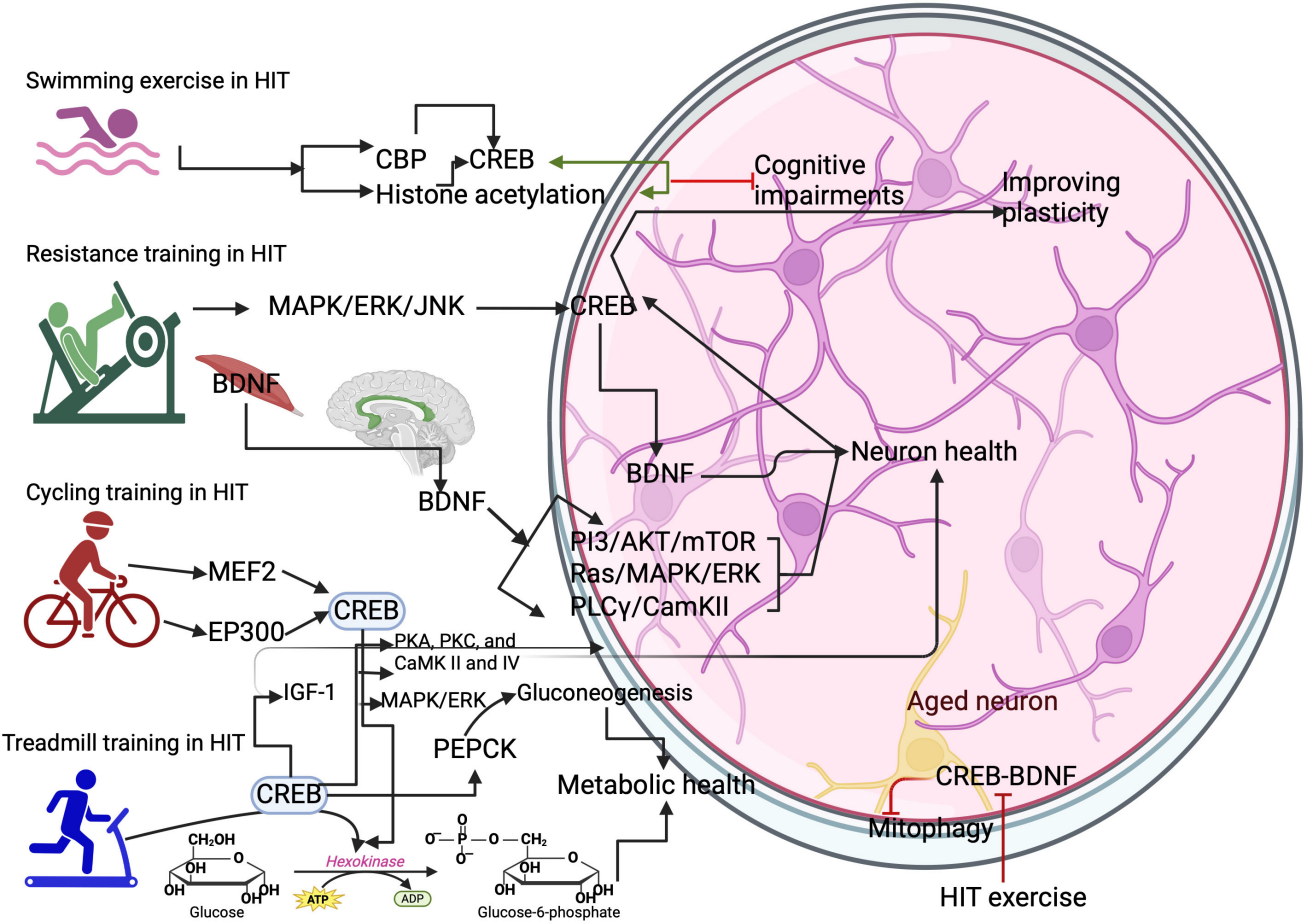
Different types of HIT exercise on activating CREB. Swimming exercise activates the CREB binding protein (CBP) and histone acetylation. This can increase the expression of CREB in the neurons. Resistance training activates the CREB through MAPK/ERK/JNK to increase BDNF-mediated benefits in the brain. Cycling training activates the myocyte enhancer factor 2 (MEF2) gene and histone acetyltransferase p300 (EP300) gene for CREB expression. Treadmill training activates the hexokinase and phosphoenolpyruvate carboxykinase (PEPCK) to improve metabolic health in the neurons. However, HIT protocols inhibit the CREB/BDNF functions and cause mitophagy in the aged neurons.

CREB is crucial for neuronal development, and studies have shown that CREB deletion leads to neurodegeneration in the hippocampus and dentate gyrus (8, 53). The possible exercise-mediated molecular cross-talk for improving neuronal survival is BDNF, insulin growth factor (IGF), pituitary adenylate cyclase-activating polypeptide, and leptin, all of which are associated with neuronal survival and development (8). However, different exercise protocols with different intensities influence the expression of these proteins. For example, HIT running protocols for 20 mins increased the activation of CREB (54), which could transcribe these proteins for neuronal survival. The voluntary running protocols increased the levels of CREB upon exercise for at least a week in the hippocampus through the activation of MAPK/ERK for improving brain functions (25) mainly; CREB could integrate several molecular pathways such as PKA, PKC, and CaMK II and IV for neuronal survival (55, 56). The possible upstream targets by HIT, such as Ca^2+^ and CaMK IV, may improve the functions of neurons by transcription of the BDNF gene mediated by CREB (57). Treadmill exercise at 90% VO2 max for 12 weeks significantly increased the IGF-1 in the serum, which enters into the hippocampus of the brain and supports the neuronal development and improves the plasticity of neurons (58), mediated by the CREB (59) (**Figure 2**). Exercise-induced leptin produces a local effect on the synapse, altering the neuronal structure and its plasticity and proliferation and differentiation through PI3/Akt, signal transducer and activator of transcription 3 (STAT3), and ERK/MAPK (60, 61) and all of these genes are converge on CREB for further their signaling activities. However, HIT with a shorter duration decreased the concentration of leptin, possibly interfering with the increase of cortisol and metabolic dyshomeostasis (61). Also, activation of inflammatory cascades by CREB can negatively contribute to brain functions, as evidenced by neuronal death (62), while the neuronal survival pathway is also activated by the CREB, such as triggering the BCl-2 genes (62). However, how this scenario is balanced in the brain function is unknown, and a study has shown that a long-term HIT program increases the BCL-2 genes in the aging model (63).

## 8 HIT-induced CREB role on brain pathology

Changes in neuronal plasticity and cognitive impairments are the main causes of brain pathology. CREB and its transcriptional cofactors are causally linked to these processes. For example, activation of CREB requires many psychiatric genes, such as AKT and MAPK3, which are mainly activated by exercise regimens. In particular, HIT exercise greatly increases the AKT (64), and MAPKs (51), which can either activate or deactivate the CREB expression (51, 64). In addition, CREB mutation and expression are linked with MEF2 and EP300 genes, which are crucial for brain development and bipolar disorders and are also increased in an exercise intensity-dependent manner. For example, aerobic exercise like running or cycling with an intensity of 70% VO2max increased the activation of MEF2C (65, 66). Exercise-induced activation of autophagy promotes neuroprotection by excluding toxic metabolites and maintaining brain tissue health, especially in long-lived neurons (12). It has been shown that CREB regulates the key autophagy genes in the neurons, mainly; HIT regulates the mTOR activity via PI3K/Akt and improves the autophagy in the hippocampus (67), and this scenario activates the CREB-mediated autophagy to induce neuroprotection in the hypoxic brain injury (68). In contrast, HIT triggers mitophagy in the aged hippocampus by disrupting the CREB and BDNF signaling (69), and this may be an exercise type-dependent effect that affects the CREB signaling in aging (70).

HIT ameliorates the Aβ accumulation-induced memory deficits (71, 72), partially mediated by CREB transactivation disruption in the AD neurons. This can improve neuroplasticity by activating CREB-mediated neuroprotective genes and memory genes via increasing BDNF expression (73, 74). Exercise from moderate to high intensity (60% to 80% VO2 max) increases peripheral blood monocyte turnover (75, 76), which can differentiate into macrophages and its secretory factors in the AD brain (74, 75). In particular, macrophage-derived factors increase neuronal survival by repairing neural damage in the nerve injury and promoting nerve regeneration (76). In this case, HIT-induced CREB regulation increases the antiinflammatory cytokines, including IL-10 (77). It increases the pro-survival signals by preventing the proinflammatory milieu to prevent neuronal loss in the AD brain (78).

## 9 Factors affecting HIT-induced CREB expression and future direction

Several factors affect HIT-induced CREB functions, which alter the molecular and cellular mechanisms related to brain health. For example, CREB-induced neurotrophic factors like BDNF require regulated expression upon HIT as it is linked with negative effects on brain health, such as inducing bipolar disorder (2). Exercise intensity during different exercise protocols alters upstream signaling pathways, such as PKA/CREB, MAPK/CREB, and CaMK/CREB pathways that lead to CREB phosphorylation. However, the specificity of these molecule’s expression is varied according to the exercise protocols. Studying other signaling pathways such as AMPK, mTOR, and hypoxia-inducible factor alpha that can cross-talk with CREB to exercise adaptation, which may effectively overcome HIT-induced initial molecular perturbation in the brain. Focusing on HIT-induced epigenetic modifications, including DNA methylation and histone acetylation, influencing the CREB activity in neurodegenerative conditions would open a new door for specific targeting therapy for neurodegenerative conditions. HIT-triggered inflammatory markers on cognitive improvements and neuroprotection following CREB upregulation may provide better insight into the role of inflammatory markers on brain health. Studies have shown that HIT alters the structural development of the brain by influencing the volume of gray matter and white matter and studying the role of CREB upon HIT will reveal how HIT-induced CREB affects communication between different brain regions (31, 79). HIT-induced CREB helps to improve various cognitive domains like memory, attention, and processing speed, and mood parameters like depression, anxiety, and stress (78), and identifying underlying mechanisms may reveal the importance of HIT-induced CREB on cognitive function and mental health. As mentioned, HIT protocols are not studied well among the aging population; evaluating the HIT protocols and exploring the extent to which HIT triggers CREB-induced neurogenesis, particularly in the aging brain, may provide insights into neuroprotective mechanisms. In addition, defining the specific intensity and duration of HIT may tailor the exercise guidelines for different populations (e.g., age groups and clinical populations). Studying individual differences like genetic factors and sex differences and identifying hormonal influences on brain health may also provide better insight into HIT and CREB expression. Moreover, finding multimodal approaches like dietary interventions and cognitive training combined with HIT protocols may promote synergistic effects on brain health. Finally, applying all these approaches to convert into translational research may effectively integrate HIT protocols into public health initiatives, clinical practices, and everyday life. Altogether, addressing these research directions can not only elucidate the comprehensive impacts of HIT-induced CREB effect on brain function but also pave the way for personalized exercise interventions to enhance cognitive health across the lifespan by targeting CREB-like proteins.

## 10 Conclusion

This review discussed the effect of HIT on CREB activation and further molecular cross-talk for improving brain health. HIT protocols increase the cross-talk of CREB`s upstream signaling, such as AKT and MAPKs. This can either activate or deactivate CREB`s phosphorylation in the brain. HIT protocols activate the metabolic genes, including hexokinase 2 and PEPCK, which can rewire the metabolic function to improve neuronal functions. Moreover, HIT influences the MEF2 and EP300 genes for brain development through CREB phosphorylation. HIT can also improve autophagy by excluding toxic metabolites in the long-lived neurons through CREB-mediated signaling, including mTOR and PI3/AKT. However, most of the studies included in this review are from treadmill exercises on CREB activation in the brain. Therefore, additional studies are warranted on the effect of other exercise types with high intensity on CREB`s functions.
