# Supplementary material for: High-intensity training on CREB activation for improving brain health: a narrative review of possible molecular talks
Source: Front Endocrinol (Lausanne). 2025 Jan 20;15:1498495. doi: 10.3389/fendo.2024.1498495 (PMC11788139; doi:10.3389/fendo.2024.1498495)
Supplement: Supplementary file 1 [file Table1.docx]

Table 1: Summary of study findings on HIT-induced CREB activation in the brain.

| Number of studies | Exercise type | Exercise duration | Brain region | Main findings | Possible molecular cross-talks | References |
| --- | --- | --- | --- | --- | --- | --- |
| 1 | Treadmilrunning | 3min of 85 % max speed and 2 min of 45 % max speed | Hipoocampus | Lactate induced CREB improved the cognitive performance, angiogeneis and metabolic flux | CREB/hormone-sensitive lipase (HSL) signaling pathway, LDH, PGC-1 alpha, SIRT1, FNDC5 and BDNF | (11) |
| 2 | Treadmill running | 8 m/min for the initial 5 min, 11 m/min for the following 5 min, and 22 m/min for the final 20 min for 8 weeks | Hippocampul | HIT had reversed effect on spatial learning and memory due to the mutual stimulation of p-CREB and BDNF mRNA | p-CREB/BDNF/NMDAR is the underlying molecular cross-talk for spatial learning and memory | (13) |
| 3 | Treadmill running |  | Hippocampul, cortex and striatum | CREB induced molecular alteration to HIT response is linked with memory disruption and altered plasticity in the brain | ERK1/2, AKT and CREB signaling and causes significant impairment in the procedural memory | (14) |
| 4 | Treadmill running | 10 m/min for the first 5 min, 15 m/min for the next 5 min, and then for the final 20 min, either 28 m/min (week 1) or 30 m/min (week 2) | Hippocampus | HIT decreased the CREB activity and negatively influce the brain function | CREB mediated miR-132 genese and BDNF signaling in the brain | (15) |
| 5 | Treadmill running | High intensity consists of day 1, 3 m/min for 30 min; day 2, 3 m/min for the initial 10 min, 6 m/min for 10 min, and 9 m/min for the final 10 min; day 3, 6 m/min for the initial 10 min, 9 m/min for 10 min, and 12 m/min for the final 10 min; and day 4 through day 14, 12 m/min for 30 min | Hippocampus | CREB were slightly increased to improve spatial learning and memory | ERK/CREB mediated BDNF activation | (16) |
| 6 | Swimming exercise | 60 min/day six days/week for six weeks. | Hippocampus | exercise promotes hypermethylation in the hippocampus and ameliorating synaptic dysfunction and cognitive impairment | PDE4 methylation activate cAMP/PKA/CREB mediated signaling in the hippocampus. | (17) |
| 7 | Running wheel exercise | six 4 min intervals at 90 % V_peak_, each interval separated by 2 min of exercise at 50 % of V_peak_ | Hippocampus | HIT disrupts the CREB and BDNF signaling after the 7-week HIIT | CREB disruption induced mitophagy signaling, such as LC3-Ⅱ, Bnip3L and disrupt mitochondrial respiratory complex due to the reduction of hippocampal plasticity | (18) |
